# An Examination of Whitewater Boaters’ Place Attachment and Specialization in Four Different River Settings

**DOI:** 10.1007/s00267-018-1082-x

**Published:** 2018-07-21

**Authors:** Silvia Kainzinger, Arne Arnberger, Robert C. Burns

**Affiliations:** 1 Institute of Landscape Development, Recreation and Conservation Planning, Department of Spatial, Landscape and Infrastructure Sciences, University of Natural Resources and Life Sciences, Vienna, Austria; 2 Recreation, Parks and Tourism Program, Division of Forestry and Natural Resources, West Virginia University, Morgantown, WV USA

**Keywords:** Place dependence, Place identity, Recreation specialization, Whitewater recreation

## Abstract

Research on place attachment suggests that place identity and place dependence differ between recreationists with varying levels of specialization, recreating in different settings and with different resource proximities to their home. To further explore this relationship, we compared place attachment and recreation specialization of whitewater boaters in four different river settings. Data were collected on three rivers in the US and one in Austria. Place attachment was measured using four place identity and four place dependence items. Recreation specialization was treated as a multivariate construct consisting of the three dimensions; behavior, skill, and enduring involvement. The results of a cluster analysis revealed three specialization clusters. Two ANOVAs were performed by using place dependence and place identity as dependent variables and specialization clusters and the sampling rivers as independent variables. Place identity was not expressed differently between rivers but differed in specialization clusters. Place dependence was different between rivers but not between specialization clusters. Findings suggest that place attachment dimensions vary in river setting and specialization levels. Management should take into account that boaters exhibit different place attachment based on the specialization level and resource proximity to their home.

## Introduction

Managing river recreation use requires information about users and their emotional bonds toward these recreational resources (Bricker and Kerstetter 2000). For several decades, natural resource agencies have been encouraged to incorporate place attachment in their decision making on managing recreation uses. Information on place attachment provide insight into the reason why people value the setting and can be used to understand stakeholders’ support or opposition to management actions and policies, and to explain conflict between stakeholder groups. Recreationists who are more attached to a resource may be affected in different ways by changes in resource management than other users (Budruk et al. 2011; Warzecha and Lime 2001; Wynveen et al. 2018). People develop attachment in the form of affective, cognitive, and conative response to interactions, and these places can become special and favorite places (Korpela et al. 2001). In the context of outdoor recreation, place attachment determines the functional and emotional-symbolic meanings people hold for a recreation setting (Schreyer et al. 1981). Several researchers have used the concept of recreation specialization to explain why people are attached to an area and found a connection between both concepts (Bricker and Kerstetter 2000; Hammitt et al. 2004; 2009; Oh et al. 2012; Williams et al. 1992). Recreation specialization is defined as a “continuum of behavior from the general to the particular, reflected by equipment and skills used in the sport and activity setting preferences” (Bryan 1977, p. 175) and is used for capturing diversity among outdoor recreationists participating in the same activity (Ditton et al. 1992).

Whitewater boaters choose a particular river for recreation based on the river difficulty among other reasons. Therefore, rivers providing different whitewater experiences attract different groups of boaters and require different management approaches to satisfy recreationists’ needs (Kainzinger et al. 2016; Warzecha and Lime 2001). Research on place attachment also found a connection with place attachment and resource proximity, whereas recreationists living in close proximity to the resource exhibited higher place attachment (Budruk et al. 2011; Kaltenborn and Williams 2002). However, past studies addressing place attachment in different river settings (Warzecha and Lime 2001) and resource proximity (Budruk et al. 2011; Kaltenborn and Williams 2002) did not include recreation specialization in their analyses. Although past research explored place attachment, recreation specialization and resource proximity in various ways, no study addressed all those three variables together. There is still a need for additional research investigating place attachment and specialization in different river settings (Bricker and Kerstetter 2000) due to inconsistent findings for the place dependence dimensions and to further explore the concepts on other rivers. Therefore, this paper compares place identity and place dependence of whitewater boaters among four rivers (three in the US and one in Austria). Each of these rivers provides different whitewater experiences. Additionally, this study explores whether whitewater boaters’ place attachment differs among specialization levels.

## Place Attachment in Outdoor Recreation

The connection between recreation areas and recreationists’ attachment to those areas has frequently been explored for various recreational activities (Bricker and Kerstetter 2000; Kyle et al. 2004a, b, c, d; Kaltenborn and Williams 2002; Warzecha and Lime 2001). A two-dimensional model, defined by the dimensions of place identity and place dependence, has often been applied to measure qualities associated with places. Place identity is conceptualized as an emotional-symbolic meaning people assign to a place (Bricker and Kerstetter 2000; Proshansky 1978; Williams and Roggenbuck 1989). It is a complex pattern of conscious and unconscious ideals, beliefs, preferences, feelings, values, goals, and skills relevant to the environment (Proshansky et al. 1983). People do not identify directly with the physical place but rather associate meanings to that place (Kyle et al. 2004b).

On the other hand, place dependence, a functional attachment, is based on the setting’s ability to facilitate the leisure experience and relates to the functional utility attributed to the setting. It is a process where people compare the quality of the current place with comparable places (Stokols and Shumaker 1981). The functional attachment is based on the area’s physical and social characteristics, and might increase if the place is in close proximity to allow for frequent visitation. Even though a place might not provide the best recreational experience, a place close to an individual’s home might still be an often used destination (Williams and Vaske 2003). Individuals might express low place dependence if they are only visitors to an area and not long-term residents (Hammitt et al. 2004). The literature also suggested other dimensions of place attachment, such as social bonding (Kyle et al. 2004a), familiarity, rootedness and belongingness (Hammitt et al. 2004). However, only a few of these additional dimensions have been integrated as often as the two-dimensional conceptualization of place attachment (Wynveen et al. 2017).

Place attachment can be linked to resource proximity (Budruk et al. 2011), and was more strongly expressed by residents of an area than visitors (Kaltenborn and Williams 2002). Forest recreationists living within 50 miles of a recreation resource had higher place identity scores compared to distant visitors (Nyaupane et al. 2003), which suggests the need for management to be aware of and responsive to proximate visitor needs. Although distant visitors identified less with resource place, they still shared an emotional attachment with it (Budruk et al. 2011).

To date, research has addressed place attachment and aimed to find explanations for different responses to place identity and place dependence. However, only one study has compared place attachment on rivers providing different experiences. Warzecha and Lime (2001) compared place attachment between Green River and Colorado River users in the Canyonlands National Park. Both rivers provide a wilderness experience and overnight trips; whereas, the Colorado River offers a more challenging whitewater trip. The researchers found higher levels of attachment of Green River users for place identity and place dependence than Colorado River users.

## Recreation Specialization of Whitewater Boaters

Past literature used the concept of specialization in terms of behavior such as length and degree of involvement as well as attitudes and values described through the centrality to an individual’s identity (Bryan 2000). We used a three-dimensional specialization construct based on the dimensions behavior, skill, and enduring involvement (McIntyre and Pigram 1992). Behavior refers to the amount and extent of participation in a recreational activity (Schreyer et al. 1984; Scott and Shafer 2001). The development of skills and knowledge is related to the experience a person has in a certain leisure activity. The longer a person participates in a leisure activity the more skill and knowledge is attained (Scott and Shafer 2001). The dimension enduring involvement is based on the sub-dimensions enjoyment, importance, self-expression and centrality, and aims to capture the affective component of specialization (McIntyre and Pigram 1992).

Individuals progress on the specialization continuum by putting effort into developing new skills and knowledge (Scott and Shafer 2001). However, progression is the exception rather than the rule (Backlund and Kuentzel 2013; Kuentzel and Heberlein 2006) and specialization does not always show a linear progression because of, for example, life-course changes (Kuentzel and Heberlein 2006).

Recreation specialization has been applied to whitewater activities to explore motivations for a specific activity (Galloway 2012), site preferences (Galloway 2012; Lee et al. 2007) as well as perceived crowding (Kuentzel and McDonald 1992; Tarrant et al. 1997; Whisman and Hollenhorst 1998). Preferences for difficult and challenging sites were related to level of specialization (Galloway 2012; Lee et al. 2007), as well as expectations, which were found to be more vague for less specialized boaters (Bricker and Kerstetter 2000; Kuentzel and McDonald 1992). The level of specialization continues to play an important role in understanding recreation behavior (Bricker and Kerstetter 2000) and to capture heterogeneity within one recreation activity (Kim and Oh 2013).

## Place Attachment and Recreation Specialization

Several researchers suggested that previous experience can increase place attachment (Hammitt et al. 2004, 2009; Williams et al. 1992). A positive relationship between place identity and frequency of participation was found for trout anglers (Hammitt et al. 2004), campers (Hammitt et al. 2009), as well as students and national park visitors (Williams and Vaske 2003). High-visit frequency was a specific indicator for place dependence for rail trail users (Moore and Graefe 1994) and urban park visitors (Eder and Arnberger 2012). However, some studies found a rather neutral or no relationship with place dependence and past experience (Bricker and Kerstetter 2000; Williams and Vaske 2003).

Oh et al. (2012) confirmed a relationship between place attachment and recreation specialization of anglers. However, they found that not all specialization dimensions were connected with place identity and place dependence. A relationship between the skill dimension and place identity was revealed, whereas the dimension commitment was positively connected with both place attachment dimensions. Place attachment was not related to behavioral dimensions of specialization. Oh et al. (2012) traced this back to a measurement issue as the item used to capture the behavioral dimension was not a measure of dependence on a fishing place.

Bricker and Kerstetter (2000) investigated the relationship of place attachment and specialization dimensions for whitewater boaters on the South Fork of the American River. Regardless of the specialization dimension, recreationists with a low level of specialization agreed less with place identity items. This can be traced back to the findings of Kuentzel and McDonald (1992), that who found lower specialized boaters had vaguer expectations about their experience. They postulated this was a result of their limited experience use history and that they had not developed attitudes, values, and beliefs about an area indicating place identity. Bricker and Kerstetter (2000) interviewed boaters on a river that provides a relatively easy whitewater experience and found that paddlers with lower ratings in the dimension skill levels were more likely to agree with place dependence. Therefore, it makes intuitive sense that paddlers with lower skill levels depend on this river to pursue this recreation activity. Past research explored place attachment and recreation specialization in various ways, but has not compared those two concepts among different river settings providing different experiences.

## Research Questions

Place attachment was found to be different among rivers with different difficulty levels (Warzecha and Lime 2001). As there is a need to explore the relationship between recreation specialization and place attachment in different river settings (Bricker and Kerstetter 2000), we developed a conceptual framework to test whether the relationship between place attachment and recreation specialization is different or stable (Table 1). Bricker and Kerstetter (2000) treated recreation specialization as a multidimensional construct individually comparing five dimensions (level of experience, skill level, centrality to lifestyle, enduring involvement, and economic investment) with place attachment. We decided to use three specialization dimensions and forming specialization groups using cluster analysis. This approach accounts for the multi-dimensionality of the recreation specialization construct (McIntyre and Pigram 1992).

**Table 1:** Difficulty level and resource proximity of the four sampling rivers

River	Difficulty level	Area
Upper Deschutes river (UD)	Difficult (class I–V)	In proximity to urban area
North Umpqua river (NU)	Intermediate (class III–IV)	Remote area
Lower Youghiogheny river (LY)	Intermediate (class III–IV)	In proximity to urban area
Salza river (SA)	Easy (class I–III)	Remote area

Rivers providing different experiences attract boaters with varying preferences and tradeoff behavior, which is valuable information for managers (Kainzinger et al. 2016). Therefore, we selected four rivers providing different whitewater recreation experiences, e.g., river difficulty, to obtain a wider range of recreation specialization across whitewater boaters. One of the sampled rivers provides a rather difficult whitewater experience with optional class V rapids, two rivers are considered intermediate and one river offers a relatively easy whitewater trip. Place attachment also differs between proximate and distant visitors (Budruk et al. 2011). We therefore selected two study rivers in remote areas and two rivers in proximity to urban areas to compare whether place attachment varies between rivers with different shares of proximate and distant recreationists.

This study expands the literature on place attachment and recreation specialization by adding the comparison of four river samples as understanding different forms of place attachment is useful to improve management of natural resources (Williams and Vaske 2003).

The following research questions guided this study:

1. RQ1: Does place identity vary based on the difficulty of the river segment? (place identity × difficulty)
2. RQ2: Does place identity vary based on the remoteness of the river? (place identity × access)
3. RQ3: Does place identity vary based on boaters’ specialization level? (place identity × specialization)
4. RQ4: Does place dependence vary based on the difficulty of the river segment? (place dependence × difficulty)
5. RQ5: Does place dependence vary based on the remoteness of the river? (place dependence × access)
6. RQ6: Does place dependence vary based on boaters’ specialization levels? (place dependence × specialization)

## Methodology

### Study Area

Data were collected on three rivers in the US, on the Upper Deschutes River, OR (UD), the North Umpqua River, OR (NU) and the Lower Youghiogheny River, PA (LY), and on the Salza River (SA) in Austria. The rivers provide different river trip experiences based on river difficulty and were located either in urban-proximate or remote areas (Kainzinger et al. 2016, 2017) (Table 1).

The UD River, located relatively close to the city of Bend, OR, is a short section of three river miles, providing whitewater from class I to III, and is highly commercially used. This section is followed by 4.5 river miles rated as class IV–V. The UD River management is overseen by the US Forest Service.. The NU, designated as a Wild and Scenic River, is located in a fairly remote area and provides whitewater recreation from class III to IV on 11 river miles. The LY River, located 70 miles southwest of Pittsburgh, PA and 40 miles northeast of Morgantown, WV, has a full allocation system managed by Pennsylvania Department of Conservation and Natural Resources. The number of people per day is limited to 960 commercial and 960 non-commercial passengers and the group size is limited to 25 people per group (Shelby and Whittaker 2008). The LY provides class III–IV whitewater recreation on 7.4 river miles. The SA is located in the state of Styria in central Austria. It provides whitewater recreation with class I–III rapids on 21 river miles (35 kilometers). A 1992 amendment to Styria state law restricts Salza River recreational rafting to groups of three or less per boat. Only registered, commercial outfitter companies with certified guides are permitted to conduct rafting tours with more than three people per boat from April 25th to October 15th. Kayaks and canoes are permitted regardless of the group size. Interpretive signs located along the river provide information about river access sites and the river use restrictions. This information is provided in eight different languages, as use from whitewater recreationists from neighboring countries has increased over the past few years.

### Data Sampling

The surveys were self-administered, and the interviewer asked the paddlers to fill out the questionnaire on-site, and took notes of the group size, user types within the groups and the survey time. Each person encountered who was willing to participate in the survey was handed a questionnaire. We used a German and an English version of the survey instrument on the SA River as some boaters on the SA did not understand German. If a person was neither able to understand the German nor English questionnaire, they were excluded from the study. The sample was stratified over weekdays and weekend days (50%:50%). Our study was limited to private boaters only, who were not using the service of a guide for this river trip.

A total number of 1096 questionnaires were collected, with 155 were conducted at the UD, 203 at the NU, 398 at the LY, and 340 at the SA. The response rate at the UD was 51%, 43% at the NU and 45% at the LY. The response rate on the SA was 84%. Paddlers on the DU, NU, and LY were approached at the boat ramp or parking lot next to the boat ramp while packing up their gear right after the trip. These river users were less willing to answer as they were exhausted from after hours of kayaking or rafting and seemingly eager to depart the recreation area. We did not find any consistent pattern of refusals (e.g., women or older adults). A non-response check asking about origin was not conducted. The narrow and steep conditions at the boat ramps on the SA made it impossible to interview paddlers on this location. Accordingly, we approached those boaters at two campgrounds in the town Wildalpen after their trip was over for the day, which resulted in a higher response rate. Out of the total number of 1096 interviews, 36 cases were not included in the ANOVA analyses due to missing values for recreation specialization and place attachment variables. Out of these 36 cases one case was from the UD data set and 35 cases belonged to the LY dataset. Therefore, the adjusted response rate for the UD is 49 and 41% for the LY.

### Questionnaire

#### Place Attachment

The questionnaire was developed by a research team that included native English and German speakers familiar with the study settings. Place attachment was conceptualized using four place identity and four place dependence items adapted from Williams and Roggenbuck (1989), Kyle et al. (2005) and Kyle et al. (2004b) (Table 2). A two-dimensional model assumes that bonds people hold for places trace back to distinct meanings (Williams and Vaske 2003). Respondents were asked to rate the eight statements on a 5 point Likert-based scale ranging from “1 = strongly disagree” to “5 = strongly agree.” Similar items have been used in previous place attachment studies (e.g., Bricker and Kerstetter 2000; Budruk et al. 2010; Eder and Arnberger 2012; Kyle et al. 2004a, b, c, d). We chose to use the two-dimensional conceptualization of place attachment as this is a frequently used approach (Wynveen et al. 2017; 2018) and because those items had been used for whitewater recreation before (Bricker and Kerstetter 2000). Additionally, we asked boaters to report their home postal code.

**Table 2:** Means of place attachment items, results of CFA and internal consistency

Place attachment items	Mean	SD	Standardized factor loadings *λ*	SE	*t*-values	*α*
*Place identity*						*.815*
“This river means a lot to me.”	4.14	.97	.768			
“I feel no commitment to this river”.^a^	3.98	1.16	.396	0.057	10.54	
“I am very attached to this river.”	3.75	1.06	.896	0.044	29.04	
“I identify strongly with this river.”	3.50	1.10	.825	0.047	26.34	
*Place dependence*						*.838*
“This river is the best place for the kind of whitewater recreation I like to do.”	3.46	1.02	.727			
“I enjoy kayaking/rafting/canoeing here more than on any other river.”	3.07	1.05	.887	0.051	24.76	
“I get more satisfaction out of visiting this river than from visiting any other river.”	3.01	1.06	.860	0.050	24.54	
“I wouldn’t substitute any other river for the type of whitewater recreation I do here.”	2.49	1.15	.569	0.055	16.17	

^a^item recoded

Scale: 1 = strongly disagree; 5 = strongly agree

#### Recreation Specialization

The conceptualization of recreation specialization was based on the theoretical foundation of the three-dimensional model suggested by McIntyre and Pigram (1992) and Scott and Shafer (2001). We treated recreation specialization as a multidimensional construct rather than a linear continuum, as this approach recognizes the conceptual and methodological multi-dimensionality of the concept (McIntyre and Pigram 1992). The variable selection was based on the approach of Oh and Ditton (2006) and McIntyre and Pigram (1992). The selected recreation specialization items were adapted to whitewater recreation similar to the study of Bricker and Kerstetter (2000). The dimension behavior was operationalized by two open ended questions: the frequency of river trips during the past 24 months without a guide in total and the frequency of river trips during the past 24 months without a guide on the sampling river (Bricker and Kerstetter 2000, Wöran and Arnberger 2012). Skill level was assessed using two questions (Bricker and Kerstetter 2000). The boaters rated their skill level on a scale of one to five (beginner, basic, intermediate, advanced, expert) and the difficulty of rapid class they felt comfortable to boat, without the service of a guide, ranging from class I to class V. The enduring involvement scale defined by four sub-dimensions (enjoyment, importance, self-expression, centrality) and presented to the respondents using eleven items on a five-point Likert scale ranging from “strongly disagree” to “strongly agree” (Bricker and Kerstetter 2000; McIntyre and Pigram 1992; Schuett 1993). Higher ratings indicate more involvement in the activity (Schuett 1993).

### Data Analyses

Confirmatory factor analysis (CFA) was used to test for the two-dimensionality of place attachment. Multiple indices assessed the goodness-of-fit between the hypothesized model and the sample data (Byrne 2010; Hu and Bentler 1999; Kyle et al. 2004b, c): Root Mean Square Error of Approximation (RMSEA), Standardized Root Mean Square Residual (SRMR), Goodness-of-Fit Index (GFI), Normed Fit Index (NFI), and Comparative Fit Index (CFI). A RMSEA value of 0.08 or less indicates a close fit to the data, values between 0.08 and 0.10 represent mediocre models. A SRMR value below 0.08 indicates a good model. Values for GFI, NFI, and CFI range from 0 to 1.00. Values of 0.90 and higher indicate that the model fits the sample data fairly well, values of 0.95 and higher indicate a well-fitting model. Within the measurement model, correlation was allowed between place identity and place dependence dimensions. Data were analyzed using AMOS. A paired sampled *t*-test was conducted to test for differences between place identity and place dependence.

The Cronbach’s alpha for place identity was .82. Place dependence showed a Cronbach’s alpha of .83. We also determined Cronbach’s alpha for each river individually to assess reliability of the sampling rivers. The Cronbach’s alpha for place identity were nearly equal for the UD (*α* = .889), NU (*α* = .842), LY River (*α* = .834) and slightly lower for the SA River (*α* = .731). A similar pattern was found for the Cronbach’s alpha for place dependence, with the rivers UD (*α* = .852), NU (*α* = .894) and LY River (*α* = .888) similar and the SA River slightly lower (*α* = .768).

In addition, we calculated how many miles boaters traveled to the sampling river based on the home postal code. We categorized boaters into two groups, whether they traveled more than 50 miles or less to the river (Nyaupane et al. 2003).

All six specialization variables were standardized to a mean of 0 and a standard deviation of 1 for subsequent analyses. We created an index out of the eleven personal commitment items by aggregating the individual item scores based on a useful Cronbach’s alpha of 0.92. Afterwards we conducted a k-means cluster analysis on the six recreation specialization items to understand the total proportion of each level of specialization (McIntyre and Pigram 1992; Oh and Ditton 2006).

We performed two 4 × 3 (River × Specialization Group) between-subject analyses of variance (ANOVA) by using place identity and place dependence as dependent variables. We also added the variable user type (kayaker, rafter, and canoer) to the model, but removed it because of unsatisfactory results. The data (*N* = 1060) violated the equal variance assumption due to significant Levene’s Test for place dependence (*p* < .001). The model for place identity resulted in a non-significant Levene’s test (*p* = .134). Both models fit the assumptions of linearity and homoscedasticity. The Kolmogorov-Smirnov test indicated that the assumption of normality is not met for the data of both models as the significance levels are below 0.05. This is due to unequal group sizes. Post hoc comparisons with the Scheffé test were performed to reveal significant differences for the main effects. To determine differences for the significant interaction term a series of t-tests was performed using Bonferroni correction.

## Results

### Sample Profile

The majority of the boaters were male on all four rivers. NU Boaters were on average older (*M* = 42.2) than boaters on the UD (*M* = 36.6), on the LY (*M* = 37.9) and on the SA (*M* = 38.2, *F*(3,1081) *=* 5.74, *p* *<* .001). More than half of all boaters indicated having at least a Bachelor’s degree. On the UD (53.5%), on the SA (77.1%) and on the LY (57.4%) most respondents were kayaking, whereas on the NU the majority was rafting (53.7%). A small percentage was canoeing on the SA (17.9%), the LY (3.3%) and the NU (1.5%, *χ*^2^ (4) = 242.316, *p* < .001). Most of the paddlers (77.5%) were repeat visitors and had been coming to the sampling rivers on average for 14 years. No differences between the four rivers were found for those two variables.

All paddlers of the three U.S. samples were from the US, and UD (78.7%) and NU boaters (80.8%) were mainly from Oregon (80.8%). LY paddlers came from Pennsylvania (41.1%) or bordering states such as Ohio, Maryland or West Virginia. Boaters on the SA River were from Austria (41.2%), Germany (34.0%) and Czech Republic (23.2%).

The postal code analysis revealed that the majority of the UD River paddlers (61%) traveled less than 50 miles from their home to the river. However, most of the boaters from the NU (97%), the SA (96%) and the LY (87%, *χ*^2^ (2) = 291.147, *p* < .001) traveled more than 50 miles from their home town for this whitewater trip.

### Place Attachment

The CFA confirmed the two-dimensionality of place attachment, although model fit was not high (*χ*^2^ (13) = 83.5, *p* < 0.001; RMSEA = 0.071; SRMR = 0.062; GFI = .973; NFI = .917; CFI = .928). An attachment index was created by aggregating the individual item scores per dimension with high values indicating high-place attachment. A moderate correlation between PI and PD was found (*r* = .338, *p* < 0.001). The corrected item-total correlations were >.50 per each dimension.

Boaters showed higher place identity to the rivers (*M* = 3.8) than place dependence (*M* *=* 3.0, *t* = 27.49, *p* < .001). Many paddlers stated that this river means a lot to them. Quite a few indicated that they would substitute another river for the type of whitewater recreation they did on this river.

### Recreation Specialization

Cluster analyses were performed for 2-, 3-, and 4-group solutions. The 3-group solution provided the best fit for the data (Table 3). The three groups approach is consistent with previous research efforts (e.g. Bricker and Kerstetter 2000; Martin 1997; Oh and Ditton 2006) and is more amenable to management (Oh and Ditton 2006). Most of the boaters were categorized as intermediate experienced (*n* = 571), followed by casual boaters (*n* = 454) and advanced boaters (*n* = 42). On the UD 36% were casual, 51% intermediate and 13% advanced paddlers. The majority on the NU were intermediate boaters (56%) followed by 43% casual and 0.5% advanced paddlers. In SA about half (51%) were casual paddlers, whereas 47% were intermediate and 2% advanced ones. Most of the LY boaters were intermediate (59%) followed by 37% casual and 4% advanced paddlers (*χ*^2^ (6) = 54.635, *p* < .001).

**Table 3:** Cluster analysis of recreation specialization

Specialization dimension	Cluster 1	Cluster 2	Cluster 3	*X*²	Cramer’s V
	*n* = 454	*n* = 571	*n* = 42		
Mean number of trips in the last 24 month without guide	Casual	Intermediate	Advanced	938.4	.663
Mean number of trips on this river in the last 24 month without guide	Casual	Intermediate	Advanced	1006.5	.687
Self-reported skill level (5 point scale)	Casual	Intermediate	Advanced	623.5	.541
Self-reported difficulty of rapid class (5 point scale)	Casual	Intermediate	Advanced	464.6	.467
Enduring involvement (5 point scale)	Casual	Intermediate	Advanced	614.3	.537

All tested specialization dimensions revealed differences across the three specialization clusters (Table 4). The higher specialized cluster was more experienced, had higher skill levels and was more involved in that activity than lower specialized clusters.

**Table 4:** Results of the specialization items across the three specialization clusters

Specialization dimension	Casual	Intermediate	Advanced	Test of sign.	
	(*n* = 454)	(*n* = 571)	(*n* = 42)		
Mean number of trips in the last 24 month without guide	8.60	45.02	222.64	*F*(2,1066) = 440.3	*p* < .001
Mean number of trips on this river in the last 24 month without guide	3.09	12.61	151.12	*F*(2,1066) = 778.6	*p* < .001
Self-reported skill level (5 point scale)	2.34	3.89	4.36	*F*(2,1066) = 545.4	*p* < .001
Self-reported difficulty of rapid class (5 point scale)	2.89	4.20	4.45	*F*(2,1066) = 339.8	*p* < .001
Enduring involvement Index???(5 point scale)	3.31	4.40	4.69	*F*(2,1066) = 525.4	*p* < .001

Differences between the rivers samples and the specialization items were found for the number of trips taken in the past 24 months in total, the number of trips in the past 24 months on the sampled rivers, the difficulty of rapid class, and enduring involvement. The variable self-reported skill level did not differ between the four rivers (Table 5).

**Table 5:** Results of the specialization items across the four rivers

	River					
Specialization dimension	UD	NU	LY	SA	Test of sign.	
	*(n* = 155)	*(n* = 203)	*(n* = 340)	*(n* = 397)		
Mean number of trips in the last 24 month without guide	55.8	40.7	32.1	21.2	*F*(3,1087) = 10.6	*p* < .001
Mean number of trips on THIS river in the last 24 month without guide	38.6	4.3	13.9	8.5	*F*(3,1088) = 34.1	*p* < .001
Self-reported skill level (5 point scale)	3.4	3.2	3.3	3.2	*F*(3,1094) = 1.2	n.s.
Self-reported difficulty of rapid class (5 point scale)	3.7	3.9	3.9	3.2	*F*(2,1078) = 33.6	*p* < .001
Enduring involvement (5 point scale)	4.0	3.9	4.1	3.8	*F*(2,1078) = 6.3	*p* < .001

### Influence of River Sample and Recreation Specialization on Place Attachment

The analysis for place identity, a 4 × 3 (River × Specialization Cluster) between-subjects ANOVA, revealed a significant main effect for specialization cluster (*F*(2,1059) = 73.35, *p* < .001, *η*² = 0.12) and a significant interaction (*F*(6,1059) = 2.46, *p* < .023). The model for place dependence showed a significant main effect for river (*F*(3,1059) = 3.14, *p* < .005) and a significant interaction (*F*(6,1059) = 3.51, *p* < .002).

Post hoc comparison with the Scheffé test revealed significant differences in response to both place attachment dimensions (Table 6). Intermediate (*M* = 4.13) and advanced boaters (*M* = 4.44) expressed higher place identity than casual paddlers (*M* = 3.49). SA paddlers were significantly less likely (*M* = 2.68) to agree with place dependence than UD (*M* = 3.16), NU (*M* = 2.87) and LY paddlers (*M* = 2.91).

**Table 6:** Adjusted means and standard deviations of place identity and place dependence for river and specialization cluster

Place attachment		River				Specialization cluster		
		UD	NU	LY	SA	Casual	Intermediate	Advanced
Place identity	*n*	154	203	363	340	452	566	42
	*M*	4.11	4.25	3.87	3.86	3.49^a^	4.13^b^	4.44^b^
	SE	.07	.27	.08	.10	.04	.04	.22
Place dependence	*n*	154	203	363	340	452	566	42
	*M*	3.16^a^	2.87^a^	2.91^a^	2.68^b^	3.07	2.98	2.68
	SE	.08	.29	.08	.11	.04	.04	.24

Rivers and specialization groups with different superscripts are significantly different at the .05 level. The *n*'s are the number of cases in each cells. Place attachment scale: 1 = strongly disagree; 5 = strongly agree

**Fig. 1:**
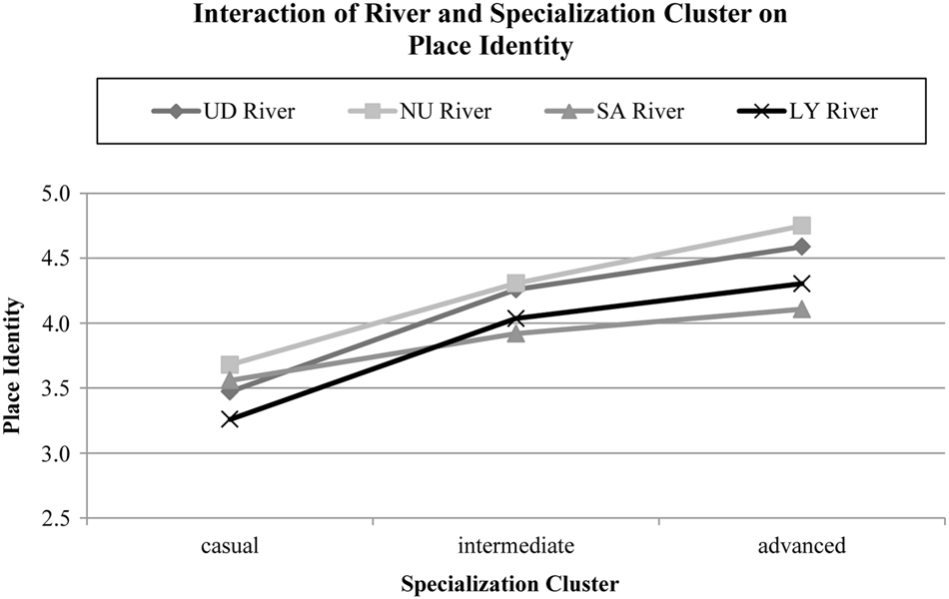
Adjusted means of place identity for river and specialization cluster

To test the interaction effects a series of *t*-tests, testing if specialization clusters differed between one river sample, revealed significant differences in response to place identity for casual and intermediate boaters in all rivers (Fig. 1). Intermediate boaters of all rivers reported higher place identity than causal boaters (Table 7). Casual and boaters on the DU and LY River expressed significantly less place identity than intermediate and advanced paddlers.

**Table 7:** Summary of group contrasts of testing the interaction of river and specialization cluster on place identity

Contrast	Group	*M*_adj_	*t*
DU river	Casual vs. intermediate	3.47	−5.68*
		4.26	
	Casual vs. advanced	3.47	−5.42*
		4.59	
	Intermediate vs. advanced	4.26	−1.67
		4.59	
NU river	Casual vs. intermediate	3.68	−5.62*
		4.31	
	Casual vs. advanced	3.68	−1.35
		4.75	
	Intermediate vs. advanced	4.31	−0.56
		4.75	
SA river	Casual vs. intermediate	3.56	−4.16*
		3.92	
	Casual vs. advanced	3.56	−1.80
		4.11	
	Intermediate vs. advanced	3.92	−0.62
		4.11	
LY river	Casual vs. intermediate	3.26	−8.89*
		4.04	
	Casual vs. advanced	3.26	−4.68*
		4.30	
	Intermediate vs. advanced	4.04	−1.22
		4.30	

*M*_*adj*_ adjusted mean place identity

*p < .05

**Fig. 2:**
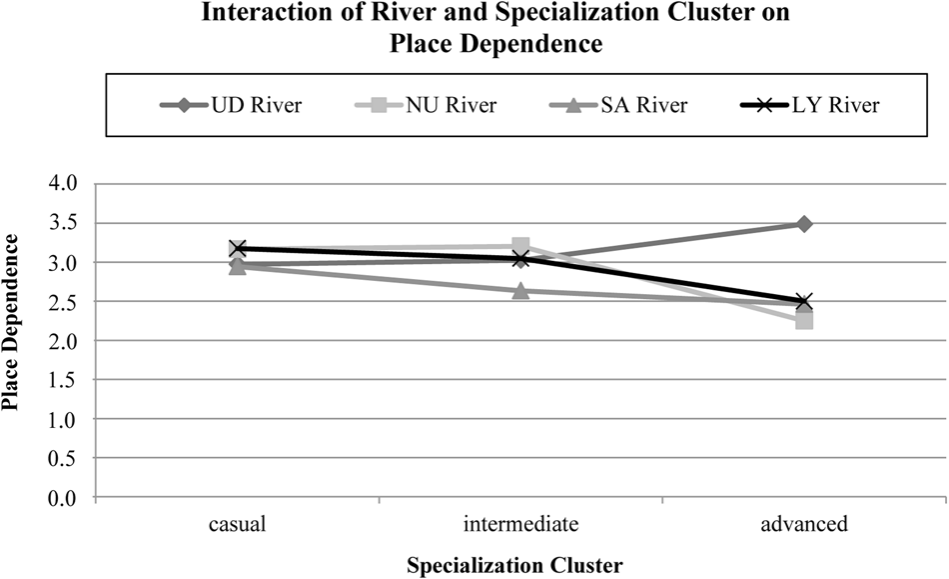
Adjusted means of place dependence for river and specialization cluster

The series of *t*-tests testing for the interaction between place dependence, is different in specialization clusters river sample, revealed that advanced boaters on the DU River reported higher place dependence than casual and intermediate paddlers (Fig. 1). On the other hand, advanced LY River paddlers reported lower place dependence than their intermediate and casual counterparts. On the SA River casual boaters expressed higher place dependence than intermediate boaters (Table 8) (Fig. 2).

**Table 8:** Summary of group contrasts of testing the interaction of river and specialization cluster on place dependence

Contrast	Group	*M*_adj_	*t*
DU river	Casual vs. intermediate	2.97	−0.35
		3.03	
	Casual vs. advanced	2.97	−2.30*
		3.49	
	Intermediate vs. advanced	3.03	−2.16*
		3.49	
NU river	Casual vs. intermediate	3.17	−0.30
		3.20	
	Casual vs. advanced	3.17	1.07
		2.25	
	Intermediate vs. advanced	3.20	1.11
		2.25	
SA river	Casual vs. intermediate	2.95	3.30*
		2.64	
	Casual vs. advanced	2.95	1.45
		2.47	
	Intermediate vs. advanced	2.64	0.17
		2.47	
LY river	Casual vs. intermediate	3.18	1.35
		3.05	
	Casual vs. advanced	3.18	2.81*
		2.50	
	Intermediate vs. advanced	3.05	2.32*
		2.50	

*M*_*adj*_adjusted mean place dependence

*p < .05

## Discussion

This study investigated the relationship between place attachment and recreation specialization among four rivers providing different whitewater experiences, and with varying proximities to urban areas. The study found differences in place attachment and recreation specialization among these rivers. Place identity did not differ between the rivers, but more specialized boaters exhibited higher place identity. Place dependence was significantly different among river settings depending more on the proximity to the resource than on difficulty of the river, but not among specialization clusters. Findings indicate that differences in the place dimensions depend on river setting and specialization cluster.

Previous literature revealed that place identity and place dependence were differently rated (Bricker and Kerstetter 2000; Kyle et al. 2003, 2004d). Our finding that boaters expressed less place dependence than place identity to the river recreation resources aligns with past findings. The literature provides several explanations for this. First, it can be traced back to measurement issues of the concept (Bricker and Kerstetter 2000; Hammitt et al. 2004). Considering the potential variety of rivers available for whitewater recreation in many areas, the place dependence items might not have addressed completely the issues of functional dependence (Bricker and Kerstetter 2000; Hammitt et al. 2004), even though the place dependence items used had shown good reliability (Williams and Vaske 2003). Boaters might feel that they have other opportunities available and are therefore less dependent. In our case this might be in particular true for LY boaters. Second, recreationists might have little dependence on the tested rivers for their desired recreational experience and other resources provide similar experiences for them (Kyle et al. 2003). Third, the majority of those boaters are visitors and not residents, which suggest place dependence might be less developed (Hammitt et al. 2004). Finally, place identity might have been rated higher, as the emotional bonds for the river reach far beyond the functionality for the whitewater activity (Bricker and Kerstetter 2000). Future research could address the differences between ratings for place dependence and place identity, using longitudinal data, exploring if emotional bonds are formed over time. Additionally, future research could integrate other place attachment dimensions such as social bonding, familiarity, rootedness or belonging (Hammitt et al. 2004; Kyle et al. 2004a) to investigate their relationships with recreation specialization more comprehensively. Past research has not yet tested the relationship of these place attachment dimensions in the case of whitewater boaters.

### Place Identity, River Setting, and Specialization Cluster

Place identity was positively related to level of specialization, confirming the results of Bricker and Kerstetter (2000) that place identity increases with level of specialization for the individual specialization dimensions. We expanded past research by combining specialization dimensions using cluster analysis showing the same pattern. Lower specialized individuals have not developed a strong combination of values, attitudes, thoughts and beliefs about an area because of their limited experience use history (Bricker and Kerstetter 2000; Kuentzel and McDonald 1992).

The place identity variable did not differ between the river settings even though we compared rivers providing different difficulty levels and located in different proximities to urban areas. In contrast, Budruk et al. (2011) found that resource proximity is an explanation for differences in place identity. Even though we selected rivers in more populated and remote areas to determine whether this might influence emotional bonds to more accessible areas, we were not able to identify differences in place identity. We identified the UD and the LY in close proximity to urban areas. Our postal code analysis revealed that the majority of the UD boaters were from the Bend, OR region, however most of the LY paddlers were not from the urban areas nearby (Pittsburgh, PA, and Morgantown, WV, respectively). It can be assumed that river users share an emotional-symbolic attachment with rivers (Budruk et al. 2011). Due to modern globalized lifestyle, people develop bonds to multiple places that extend over large geographical areas (Kaltenborn and Williams 2002) and are not just located in a close radius to their home. Our results also demonstrated this in an international context, as we did not find any differences in place identity between the US rivers and the river in Austria. The question remains open whether whitewater boaters’ place identity is similar for all river settings in the western context. Our findings also contradict the results of Warzecha and Lime (2001), who compared rivers in the same national park with varying difficulty levels. We assume that other variables besides river difficulty have to be integrated in a model to be able to explain differences in place identity among river settings. Future research may investigate the relationship between place identity and other variables such as ideals, beliefs, values, goals, and motivations among different settings.

Differences among river settings might be explained by looking at the results for place identity by controlling for river and specialization cluster. Casual boaters of the UD and LY River expressed less place identity than their intermediate and advanced counterparts. Intermediate and advanced boaters had higher ratings on the dimensions of skill level and personal commitment than casual paddlers. Skill level and personal commitment were positively related with place identity in the case of anglers and whitewater boaters (Bricker and Kerstetter 2000; Oh et al. 2010, 2012). It seems that UD and LY boaters, with higher commitment, gradually develop preferred identities to places (Oh et al. 2012). However, on the SA and NU differences were only found between casual and intermediate boaters. Those differences can be explained, as it takes time to develop identity (Bricker and Kerstetter 2000). It may also be possible that this is a result of the insufficient numbers of advanced boaters on those river settings due to the cluster analysis, what can be seen as a limitation of this study.

### Place Dependence, River Setting and Specialization Cluster

Similar to past research (Bricker and Kerstetter 2000; Hammitt et al. 2004; Kyle et al. 2003) the mean ratings of place dependence showed “neutral” response, indicating that boaters neither agreed nor disagreed with the feeling of being dependent on this particular resource. The analyses revealed significant differences between the SA boaters and the three US samples. SA boaters disagreed with place dependence, while boaters in the US samples rated it neutrally. The place dependence dimension is more site specific than place identity. The majority of the NU, LY, and SA boaters traveled more than 50 miles to the river, but the SA boaters exhibited lower place dependence. The low place dependence of SA boaters might be explained by looking at their origins. About half of the SA boaters (55%) were from neighboring countries, such as Germany and Czech Republic. Therefore, the SA River was not in close proximity to their home and for about more than half of the paddlers even in a different country. Boaters from those neighboring countries took fewer trips to the SA as the Austrian boaters, and may not have developed place dependence to the resource (Hammitt et al. 2004).

Differences in place dependence among the UD, NU, and LY can be explained by looking at the results when controlling for specialization cluster and river setting. Advanced UD boaters were more dependent on the UD River as their intermediate and casual counterparts. The majority of these boaters lived near Bend, OR, in close proximity to the river, meaning they were not just visitors, but long-term residents having developed higher place dependence (Hammitt et al. 2004). The UD River was their first choice of river to go to participate in the whitewater activity. This argument is also proven as their number of trips taken on the UD River was relatively high—visiting almost daily. High-visit frequency is a positive indicator for place dependence (Eder and Arnberger 2012; Moore and Graefe 1994). Our results lead to the conclusion that boaters who are highly specialized and who are living close to a river providing challenging whitewater experience are likely to develop strong place dependence to this river resource.

Conversely, advanced LY boaters lived further away from this river and expressed lower place dependence than their casual and intermediate counterparts. Those boaters might have multiple challenging rivers and streams available for whitewater boating in a reasonable distance, and the sampled river does not engender any special relationship for them (Bricker and Kerstetter 2000). Similar to the advanced UD boaters, advanced LY boaters visited on average twice as many rivers over the past 24 months than the sampled river. As the LY River was not close to their home, they may depend less on it.

The majority of casual boaters were found at the SA River. This river provides a relatively easy whitewater experience and it seems to be logical that boaters with lower skill level choose the SA River. They were more dependent on this river than intermediate and advanced boaters—probably because the SA River was too easy for them. A similar finding for the skill dimension was reported by Bricker and Kerstetter (2000) for a river providing a relatively easy whitewater experience. It appears that place dependence for rivers with lower difficulty levels seems to be consistently higher for boaters with lower skill levels.

Place dependence did not differ among specialization clusters, which aligns with the findings of Bricker and Kerstetter (2000), who only found significant results for two out of five specialization dimensions. Our findings show that the relationship of place dependence and recreation specialization can be explained by taking into account river setting. Rivers providing opportunities for more challenging whitewater recreation attract more advanced boaters who can develop higher place dependence. Boaters on the SA River were less specialized and exhibited lower place dependence, in comparison to NU and LY paddlers who were more specialized and showed higher place dependence.

## Management Implications and Conclusions

This study compared place attachment and recreation specialization across four river settings providing different difficulty levels and varying resource proximities. Study findings represent insights into differences and commonalities in place identity and place dependence based on level of specialization and river setting. Our results did not show distinct differences in either concept across the four river settings, in particular between the US and Austria. However, our results indicated that the relationship between place attachment, river setting and specialization level was visible by controlling for river setting and specialization level. Whitewater boaters tend to choose a river based on their preferred conditions and skill levels (Kainzinger et al. 2016; Lee et al. 2007). We were able to explain our results with differences based on the specialization dimensions treated as a multidimensional construct (McIntyre and Pigram 1992). Skill level and personal commitment influenced place identity (Oh et al. 2010, 2012), whereas frequency of participation (Eder and Arnberger 2012; Smaldone et al. 2005) was an indicator of place dependence.

It appears that whitewater recreationists in the US and Austria have similar place identities associated with rivers used as a recreation resource. Study results on differences in reliability of the place attachment dimensions across German and English speaking countries confirm results reported by Wynveen et al. (2017). It might also be difficult to infer cultural differences, as we did not specifically integrate variables to be able to compare cultural differences. Further research is necessary to see if this holds true for other recreational activities and to integrate other countries in the discussion as well (Budruk 2010).

Our results revealed that place dependence differed between the river settings, and we were able to explain those differences by controlling for specialization cluster and with resource proximity. Our findings for the UD River show that rivers located near a larger city are used more frequently for recreational boating, and that those boaters living in close proximity also depend on this river in particular for whitewater recreation. There seems to be not only a correlation between place identity and proximity (Budruk et al. 2011) but also between place dependence, specialization level and resource proximity to home.

Our findings also point out several management implications. Managers should seek input from the highly specialized recreationists living in the local community as they may be very valuable and knowledgeable stakeholders. Management should provide opportunities for local residents to engage in events that foster an understanding of place attachment (Budruk et al. 2011), as those recreationists may be highly affected by changes to the use of the resource. Place attachment can also contribute valuable information to management decisions. This includes the use of planning frameworks that recognize the potential value of managing a resource to provide more than one type of recreation opportunity (e.g., the Recreation Opportunity Spectrum (ROS) developed by Clark and Stankey (1979).

In this case, the larger amount of non-local whitewater boaters at the NU, LY, and SA rivers suggests they may not be resource dependent, as ratings for place dependence were almost neutral. This suggests these boaters may have multiple choices for resources for whitewater boating (Bricker and Kerstetter 2000). From a management perspective, this indicates non-local whitewater boaters might have other motivations, such as challenge (Galloway 2012), rather than the whitewater location itself. Resource managers should also fully understand potential users to their rivers, which can allow whitewater boaters choose a river based on their preferences and skill levels (Kainzinger et al. 2016, 2017).

To encourage resource managers to fully understand recreationists, it may be essential to address recreation specialization together with place attachment to discover the emotional values people hold with a recreation resource. With our multi-river study we were able to confirm findings of past research of Bricker and Kerstetter (2000) on the relationship between place attachment and recreation specialization of whitewater boaters. It appears that the positive relationship between place identity and recreation specialization is consistent for water-based recreation resources. However, the negative relationship between place dependence and recreation specialization has to be explored further by integrating additional variables, such as proximity to home to the model. Place attachment and recreation specialization continue to be important indicators to understand recreation behavior.

When viewed in a holistic manner, this unique study of four whitewater rivers has the potential to provide managers with valuable information that can be used to better understand the desires of a broad array of recreationists participating in a unique setting. As noted by Shafer (1969) there is no “average” recreationist—resource managers must attempt to meet the needs of recreationists who desire to participate in specific types of recreation. Additionally, making use of these constructs can provide managers a goal to work toward, as suggested by Burns and Graefe (2006). Although managers cannot achieve all of the goals to which they aspire, they can continually improve and make resource decisions based on scientific findings that will be more defensible when challenged (Burns and Graefe 2006).
